# Supplementary figures and images for: Genome-wide identification of gene families related to miRNA biogenesis in Mangifera indica L. and their possible role during heat stress
Source: PeerJ. 2024 Jul 17;12:e17737. doi: 10.7717/peerj.17737 (PMC11260077; doi:10.7717/peerj.17737)

**A**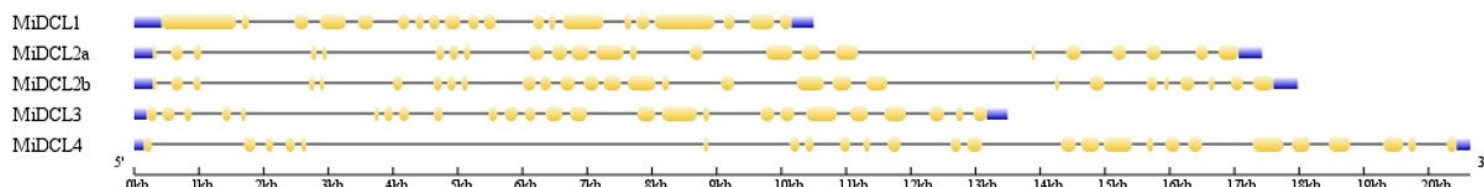**B**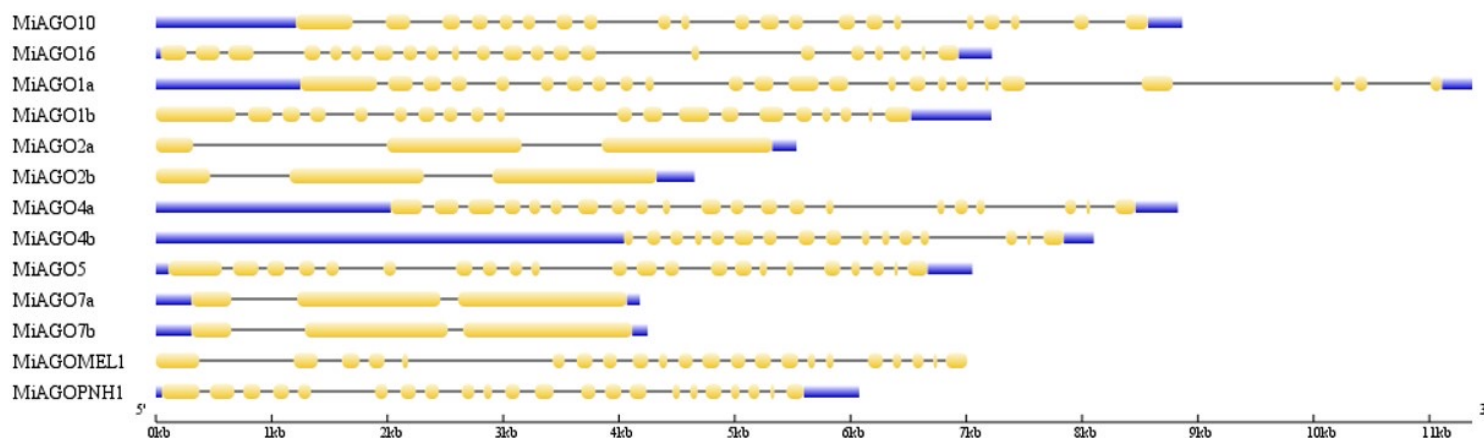**C**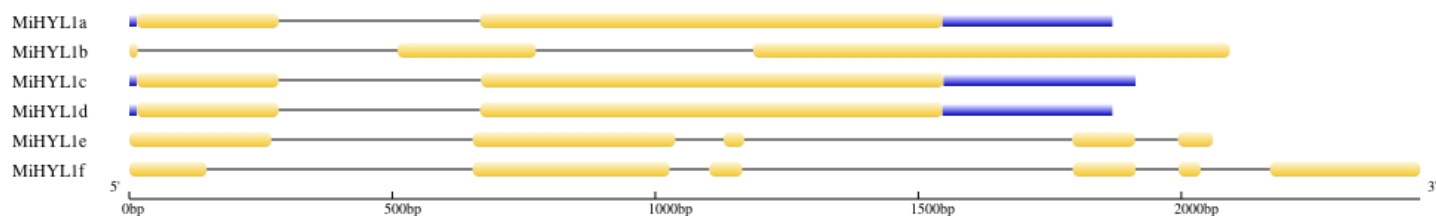**D**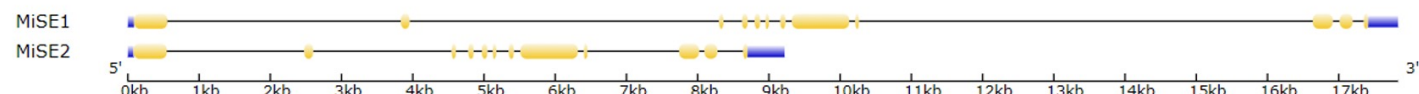**E**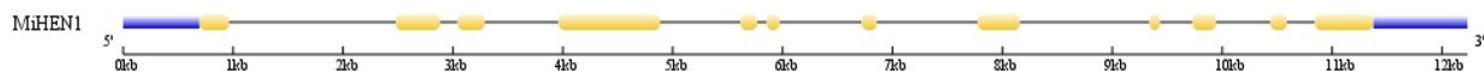**F**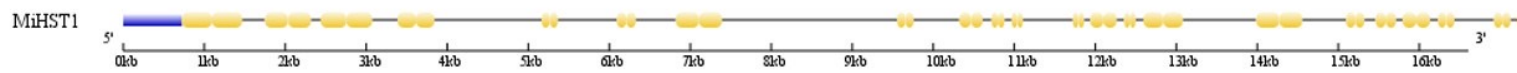**G**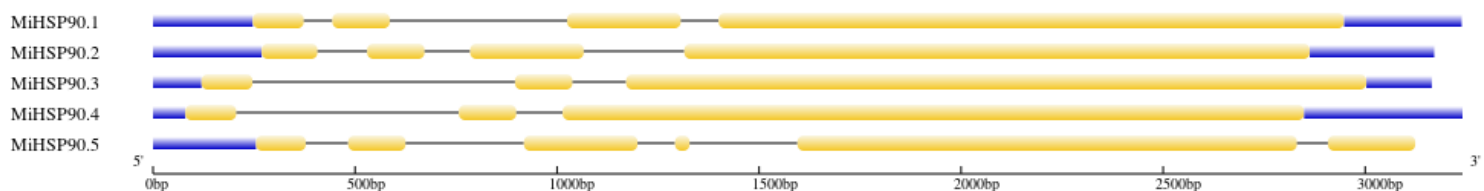

Legend:

 CDS
  upstream/ downstream
  Intron

Supplement: Supplemental Information 1 — Exons are indicated in yellow, introns in black, and non-coding UTR regions in blue. [file peerj-12-17737-s001.pdf]
