# Supplementary material for: Genome-wide identification of gene families related to miRNA biogenesis in Mangifera indica L. and their possible role during heat stress
Source: PeerJ. 2024 Jul 17;12:e17737. doi: 10.7717/peerj.17737 (PMC11260077; doi:10.7717/peerj.17737)
Supplement: Supplemental Information 2 [file peerj-12-17737-s002.pdf]

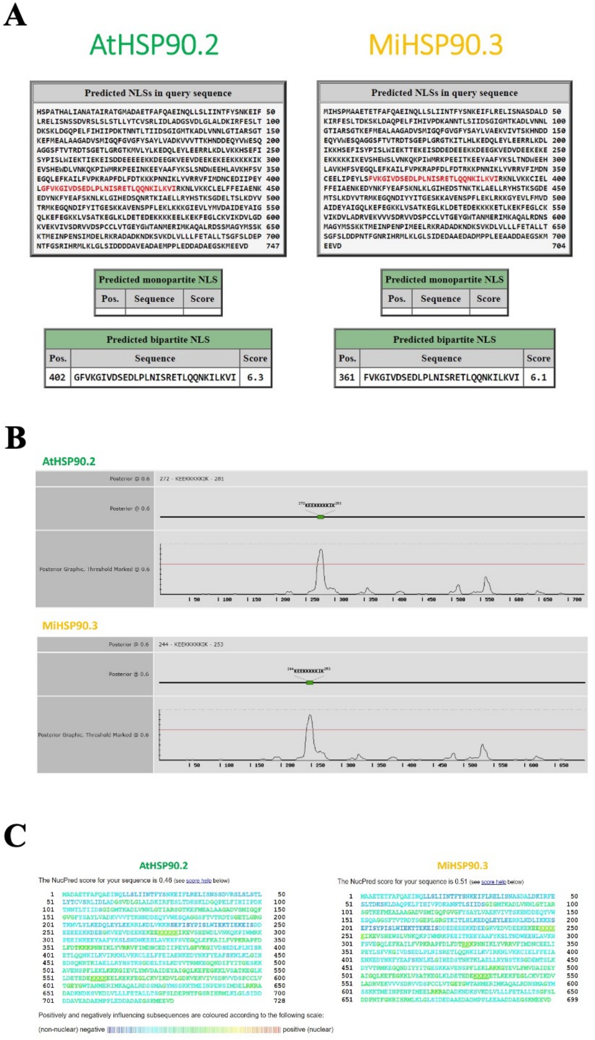
A
AtHSP90.2
MiHSP90.3
Predicted NLSs in query sequence
Predicted monopartite NLS
Pos. Sequence Score
Predicted bipartite NLS
Pos. Sequence Score
402 GFVKGIVDSEDLPLNISRETLQQNKILKVI 6.3
361 FVKGIVDSEDLPLNISRETLQQNKILKVI 6.1
B
AtHSP90.2
Posterior @ 0.6
272 - KEEKKKKKIK - 281
Posterior Graphic. Threshold Marked @ 0.6
MiHSP90.3
244 - KEEKKKKKIK - 253
C
AtHSP90.2
MiHSP90.3
The NucPred score for your sequence is 0.46 (see score help below)
The NucPred score for your sequence is 0.51 (see score help below)
Positively and negatively influencing subsequences are coloured according to the following scale:
(non-nuclear) negative
positive (nuclear)
