# Supplementary material for: Genome-wide identification of gene families related to miRNA biogenesis in Mangifera indica L. and their possible role during heat stress
Source: PeerJ. 2024 Jul 17;12:e17737. doi: 10.7717/peerj.17737 (PMC11260077; doi:10.7717/peerj.17737)
Supplement: Supplemental Information 3 — Colored boxes indicate characteristic domains. [file peerj-12-17737-s003.pdf]

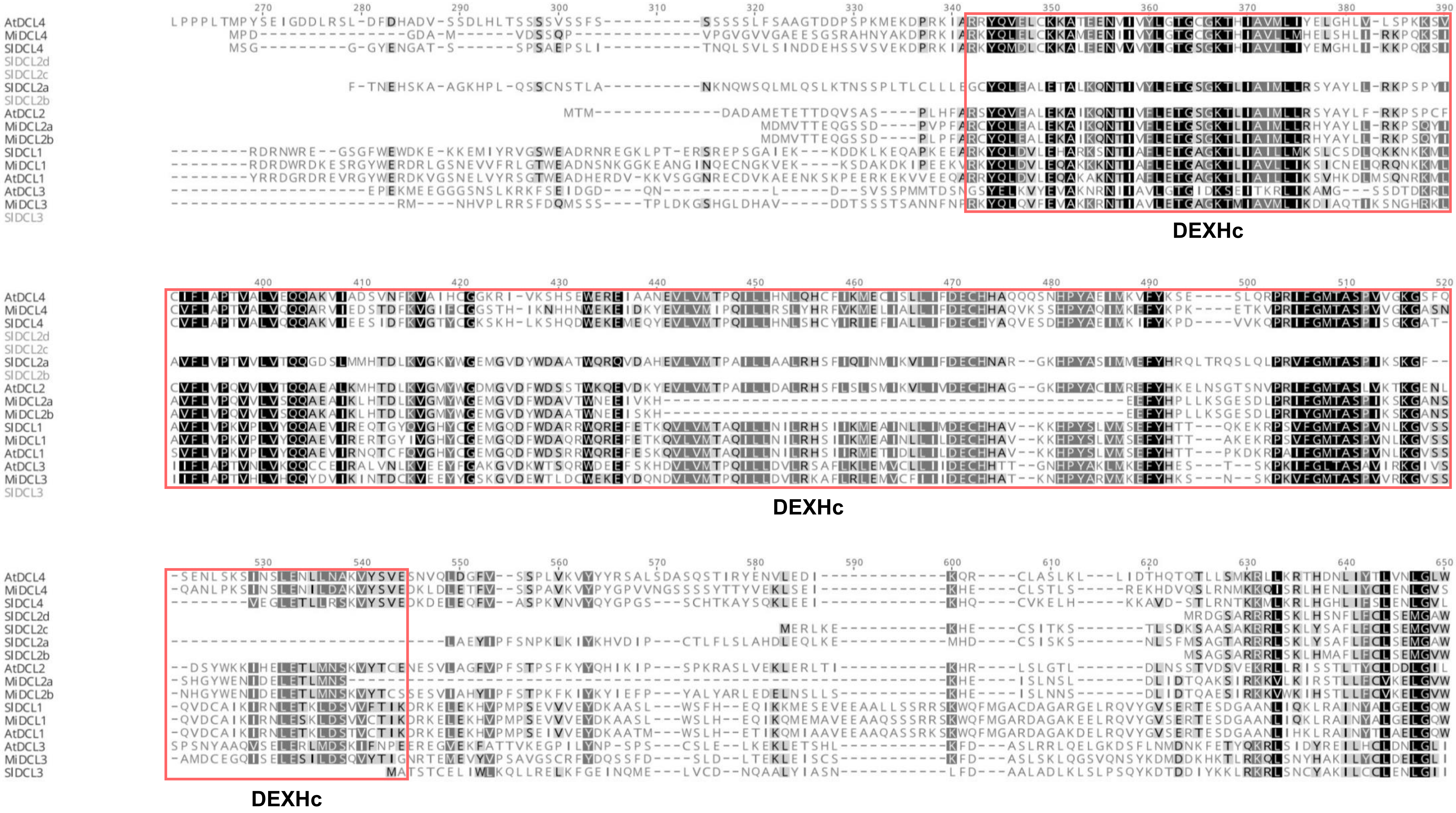
AtDCL4
MiDCL4
SlDCL4
SlDCL2d
SlDCL2c
SlDCL2a
SlDCL2b
AtDCL2
MiDCL2a
MiDCL2b
SlDCL1
MiDCL1
AtDCL1
AtDCL3
MiDCL3
SlDCL3
DEXHc
DEXHc
DEXHc

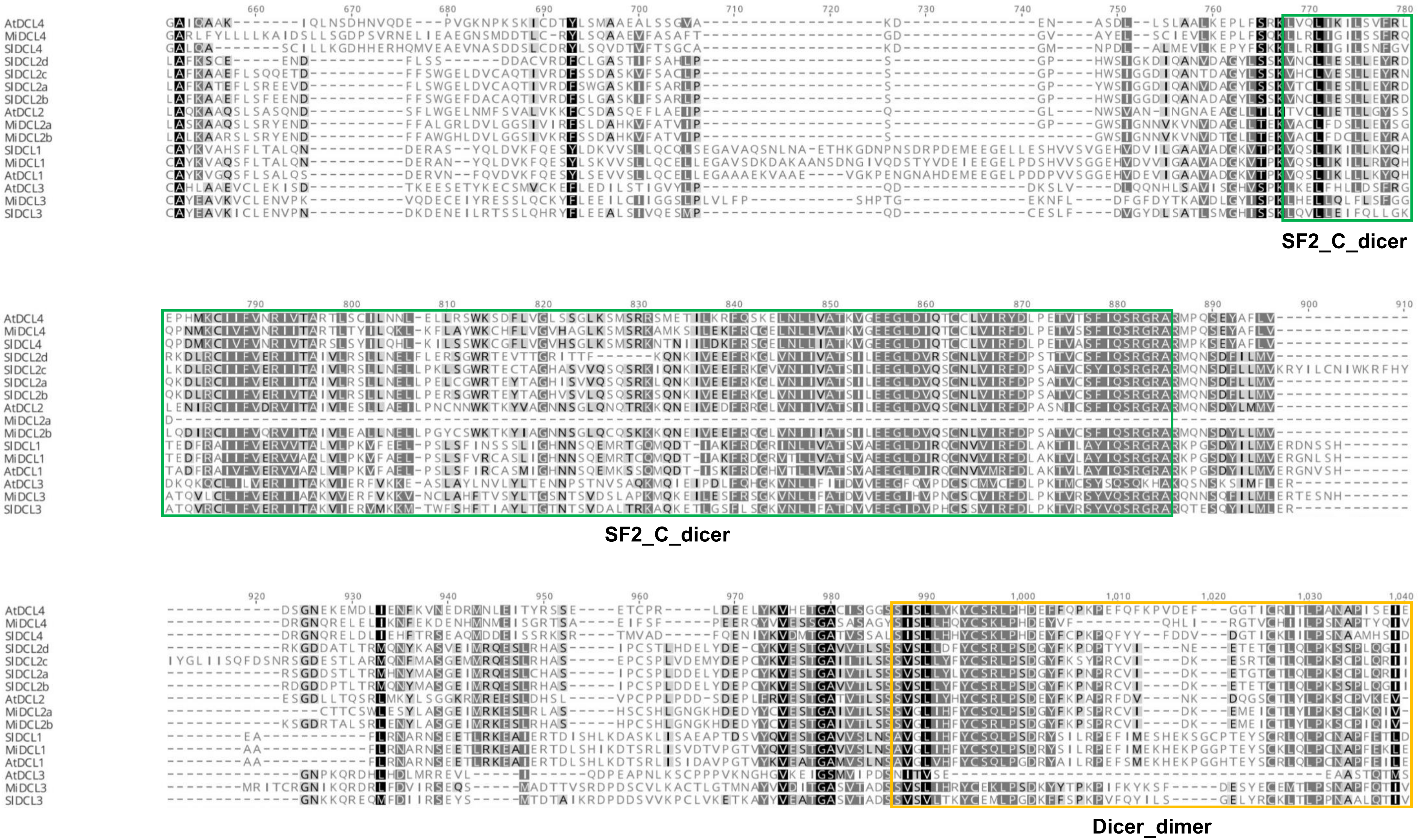
SF2_C_dicer
SF2_C_dicer
Dicer_dimer

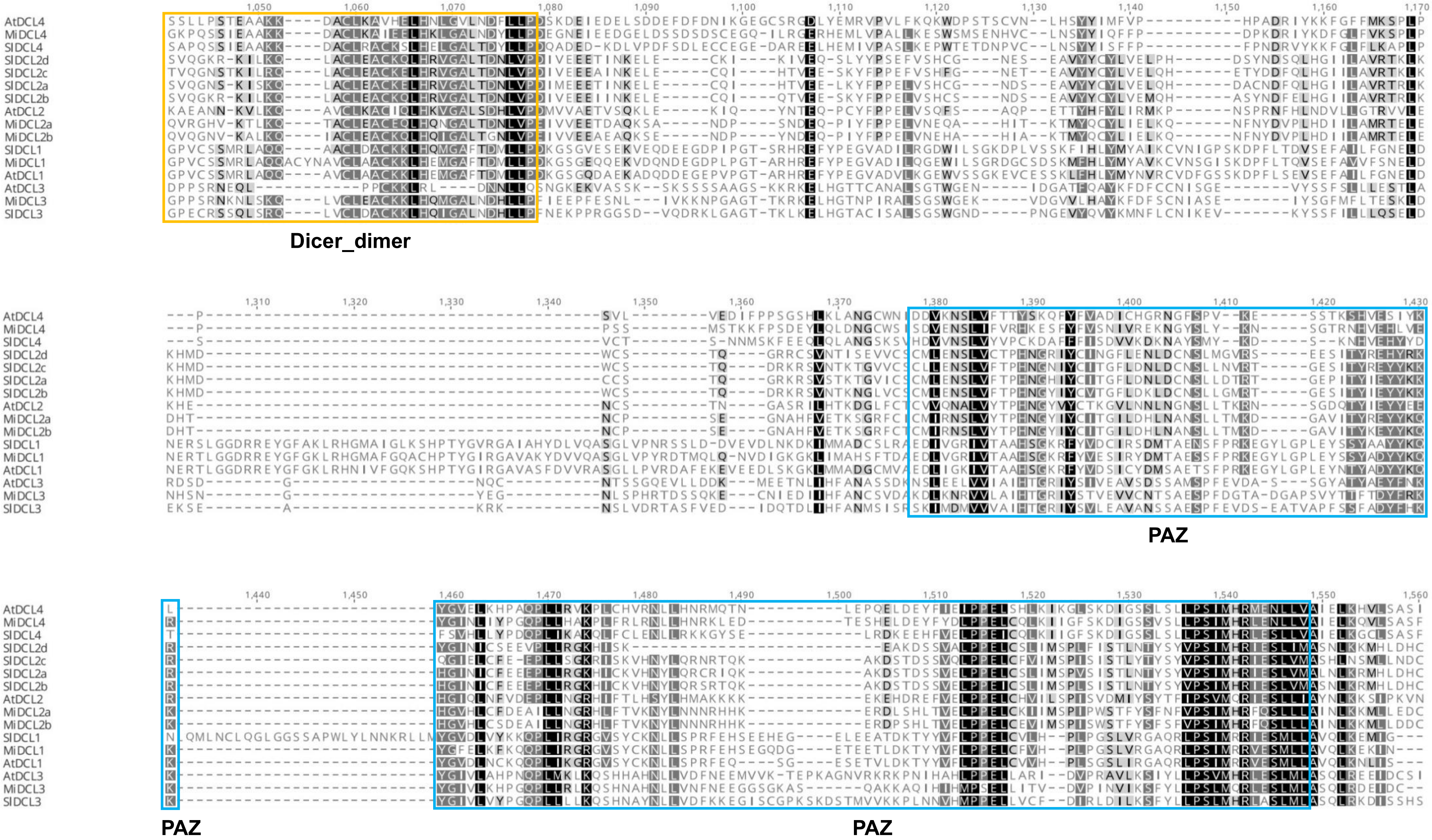

AtDCL4
MiDCL4
SlDCL4
SlDCL2d
SlDCL2c
SlDCL2a
SlDCL2b
AtDCL2
MiDCL2a
MiDCL2b
SlDCL1
MiDCL1
AtDCL1
AtDCL3
MiDCL3
SlDCL3
Dicer_dimer
PAZ
PAZ
PAZ

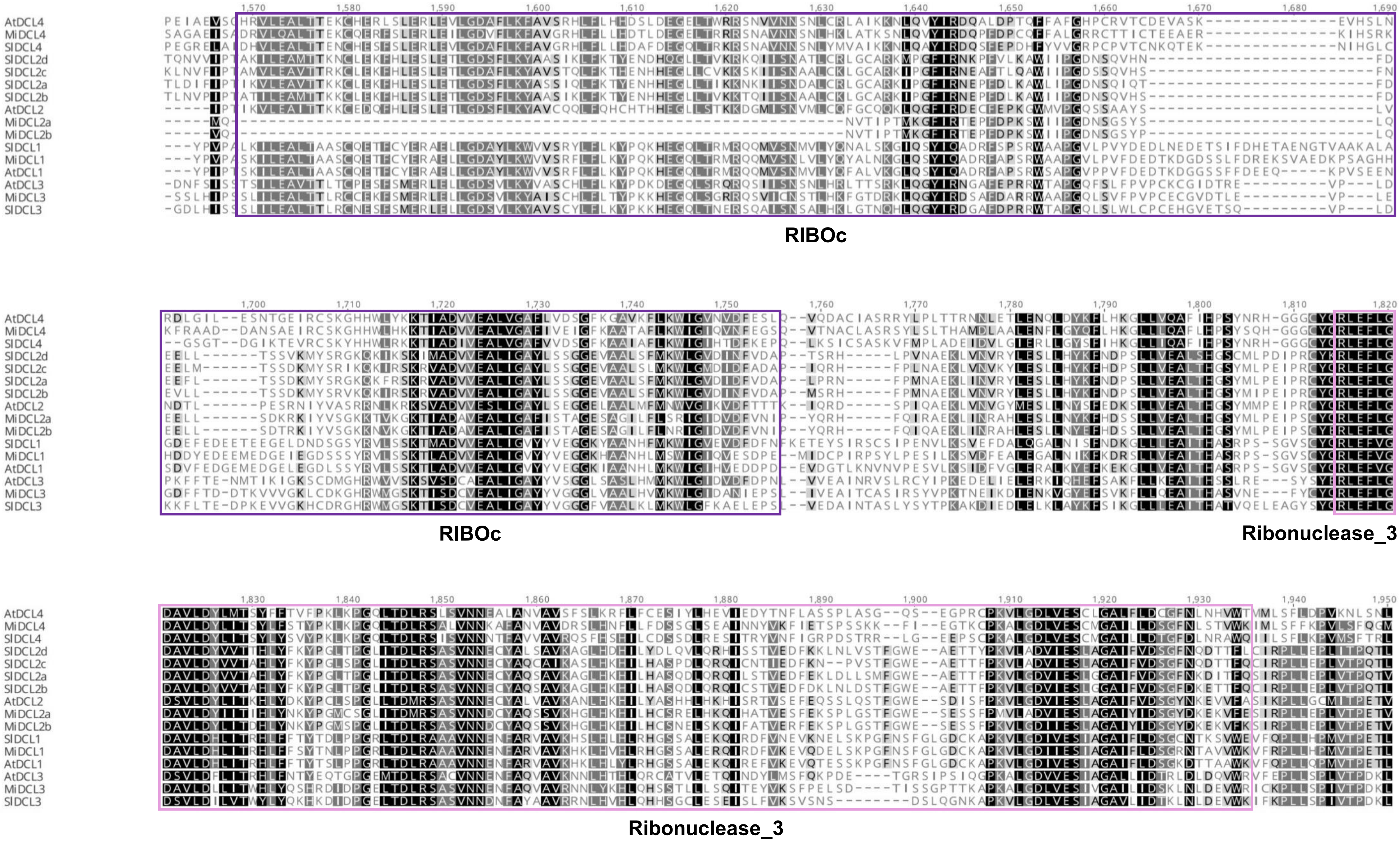
AtDCL4
MiDCL4
SlDCL4
SlDCL2d
SlDCL2c
SlDCL2a
SlDCL2b
AtDCL2
MiDCL2a
MiDCL2b
SlDCL1
MiDCL1
AtDCL1
AtDCL3
MiDCL3
SlDCL3
RIBOc
RIBOc
Ribonuclease_3
Ribonuclease_3

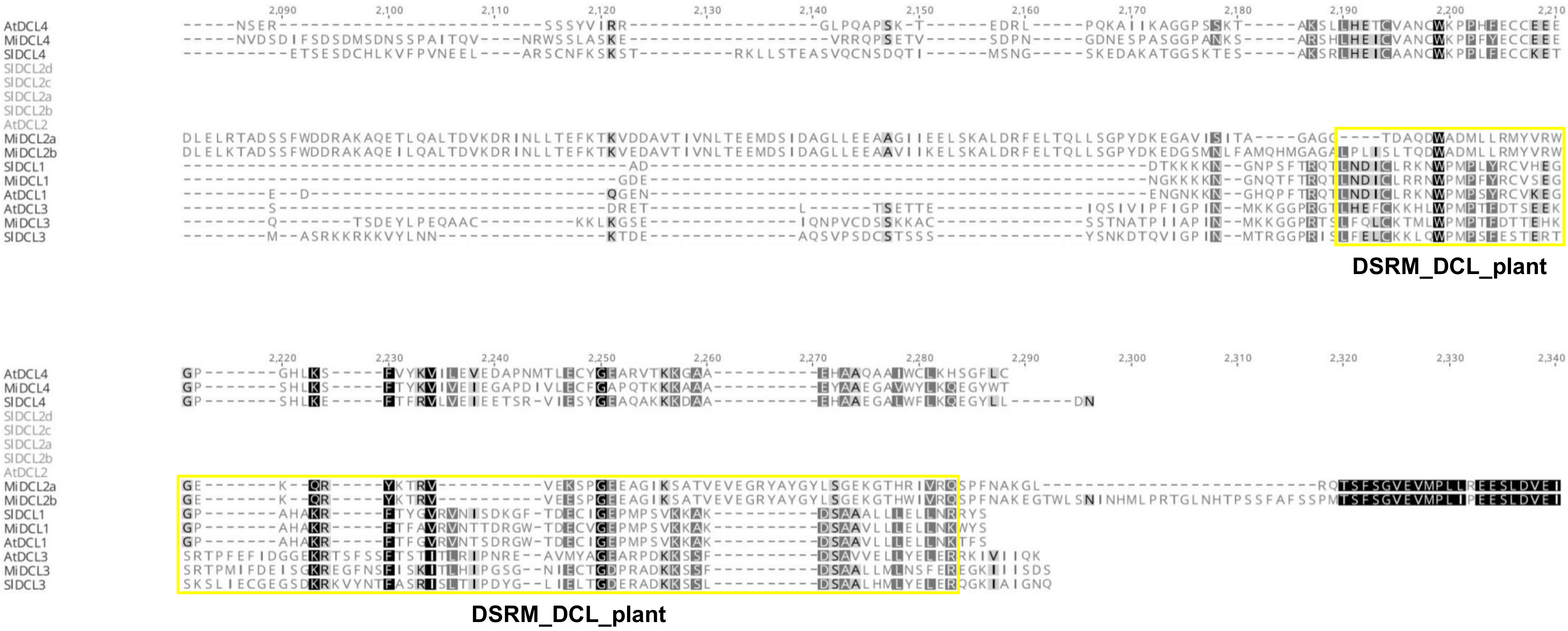
AtDCL4
MiDCL4
SlDCL4
SlDCL2d
SlDCL2c
SlDCL2a
SlDCL2b
AtDCL2
MiDCL2a
MiDCL2b
SlDCL1
MiDCL1
AtDCL1
AtDCL3
MiDCL3
SlDCL3
DSRM_DCL_plant
DSRM_DCL_plant
