# Supplementary material for: Genome-wide identification of gene families related to miRNA biogenesis in Mangifera indica L. and their possible role during heat stress
Source: PeerJ. 2024 Jul 17;12:e17737. doi: 10.7717/peerj.17737 (PMC11260077; doi:10.7717/peerj.17737)
Supplement: Supplemental Information 4 — Colored boxes indicate characteristic domains. [file peerj-12-17737-s004.pdf]

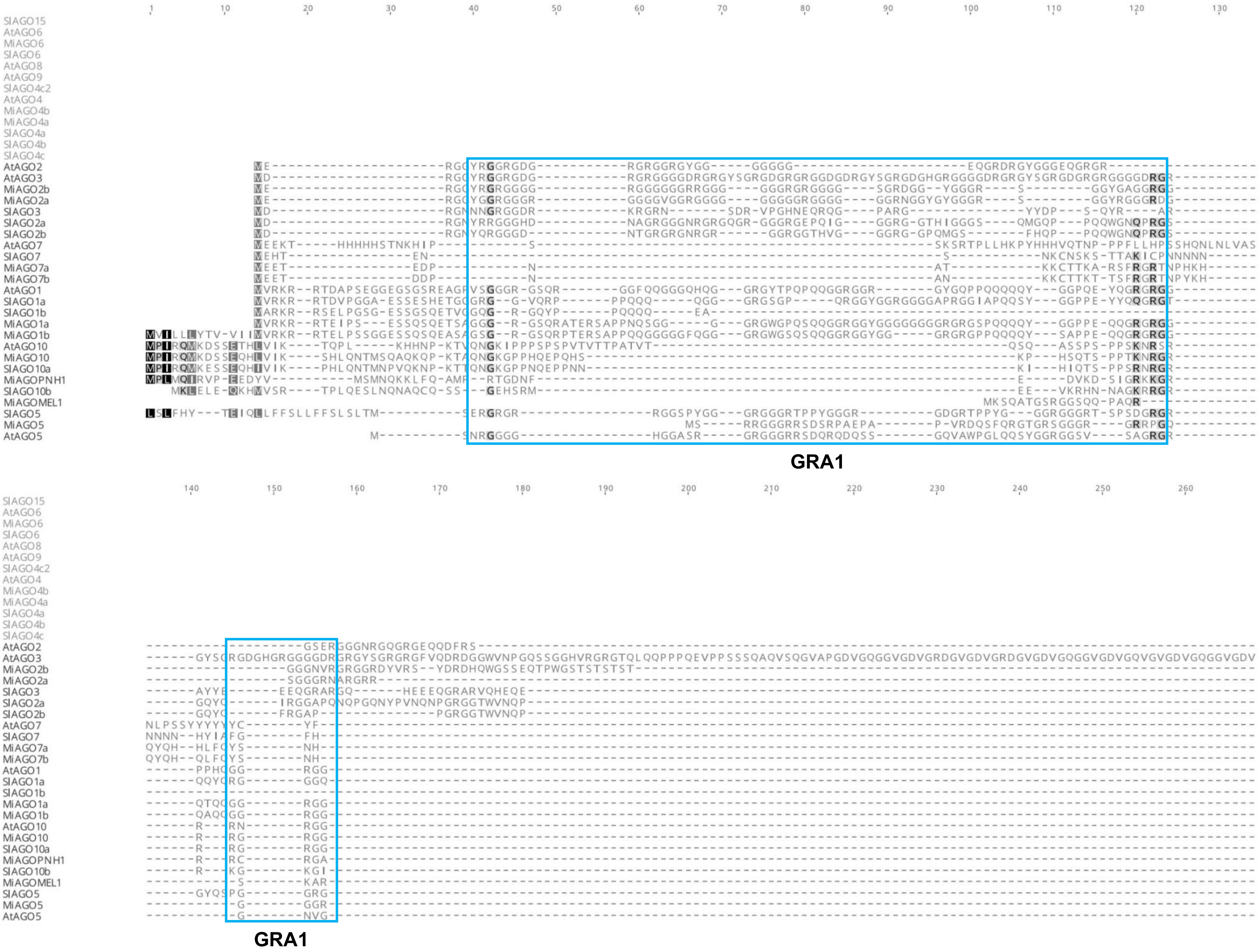

1 10 20 30 40 50 60 70 80 90 100 110 120 130
SlAGO15
AtAGO6
MiAGO6
SlAGO6
AtAGO8
AtAGO9
SlAGO4c2
AtAGO4
MiAGO4b
MiAGO4a
SlAGO4a
SlAGO4b
SlAGO4c
AtAGO2
AtAGO3
MiAGO2b
MiAGO2a
SlAGO3
SlAGO2a
SlAGO2b
AtAGO7
SlAGO7
MiAGO7a
MiAGO7b
AtAGO1
SlAGO1a
SlAGO1b
MiAGO1a
MiAGO1b
AtAGO10
MiAGO10
SlAGO10a
MiAGOPNH1
SlAGO10b
MiAGOMEL1
SlAGO5
MiAGO5
AtAGO5
GRA1
140 150 160 170 180 190 200 210 220 230 240 250 260
GRA1

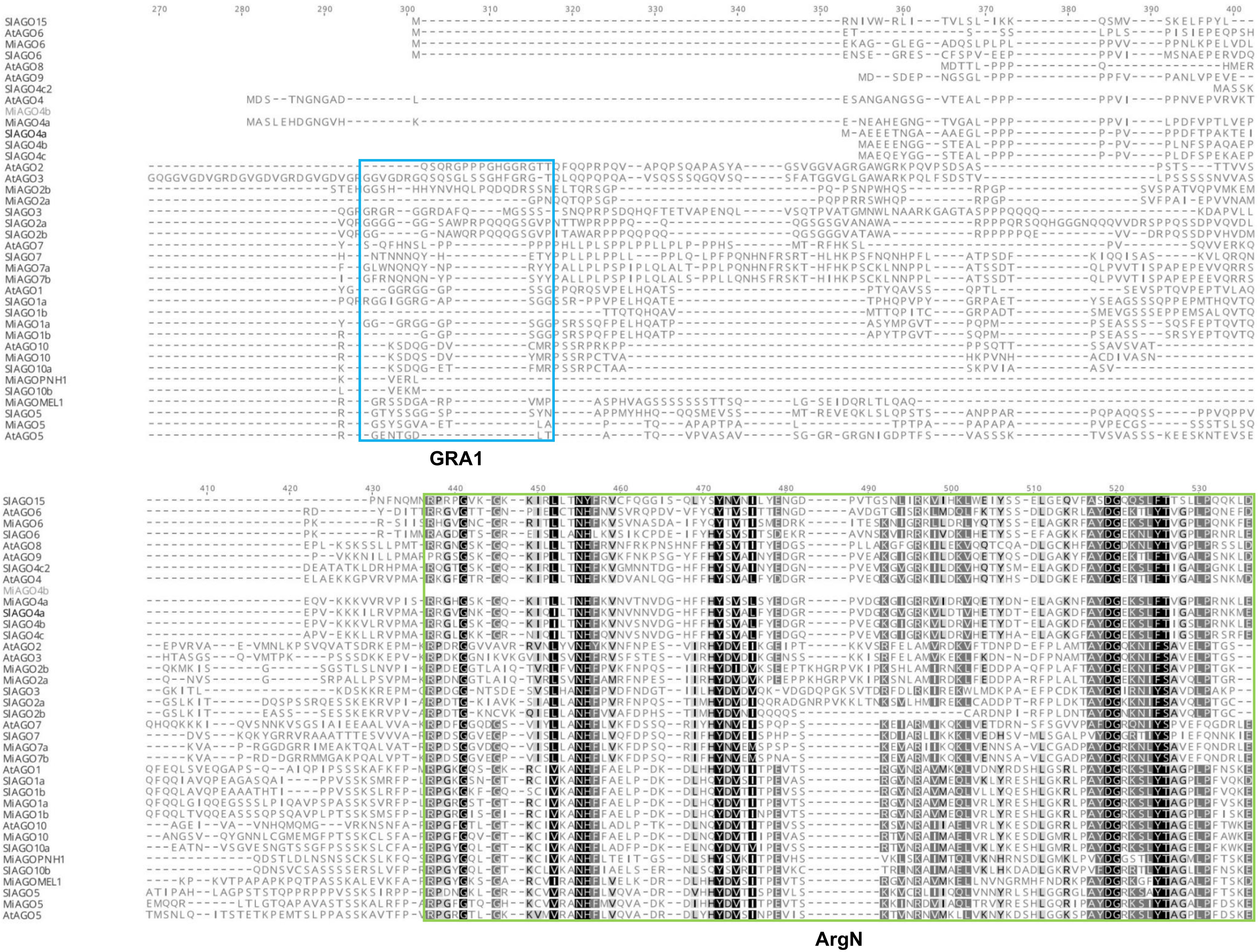
GRA1
ArgN

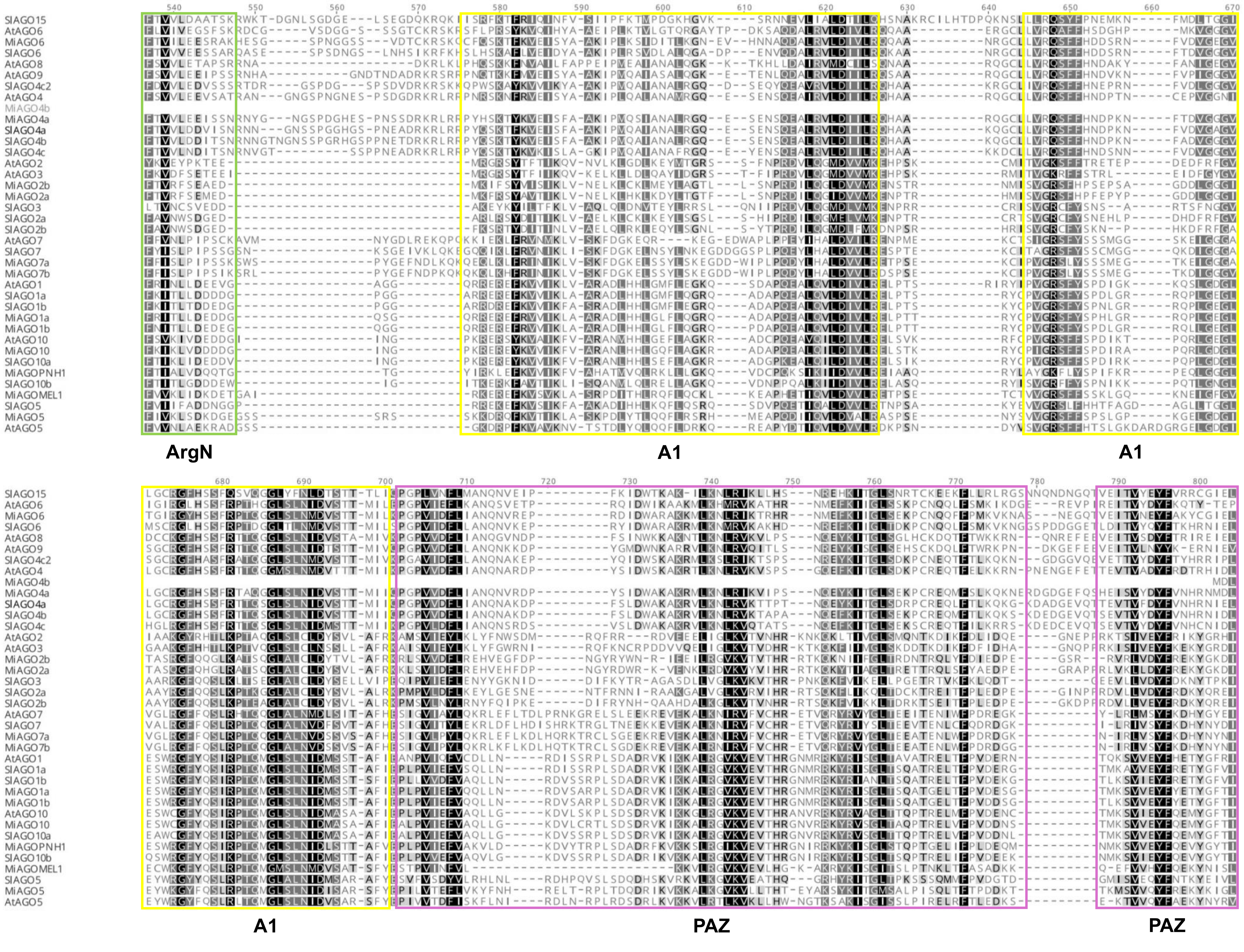
ArgN
A1
A1
A1
PAZ
PAZ

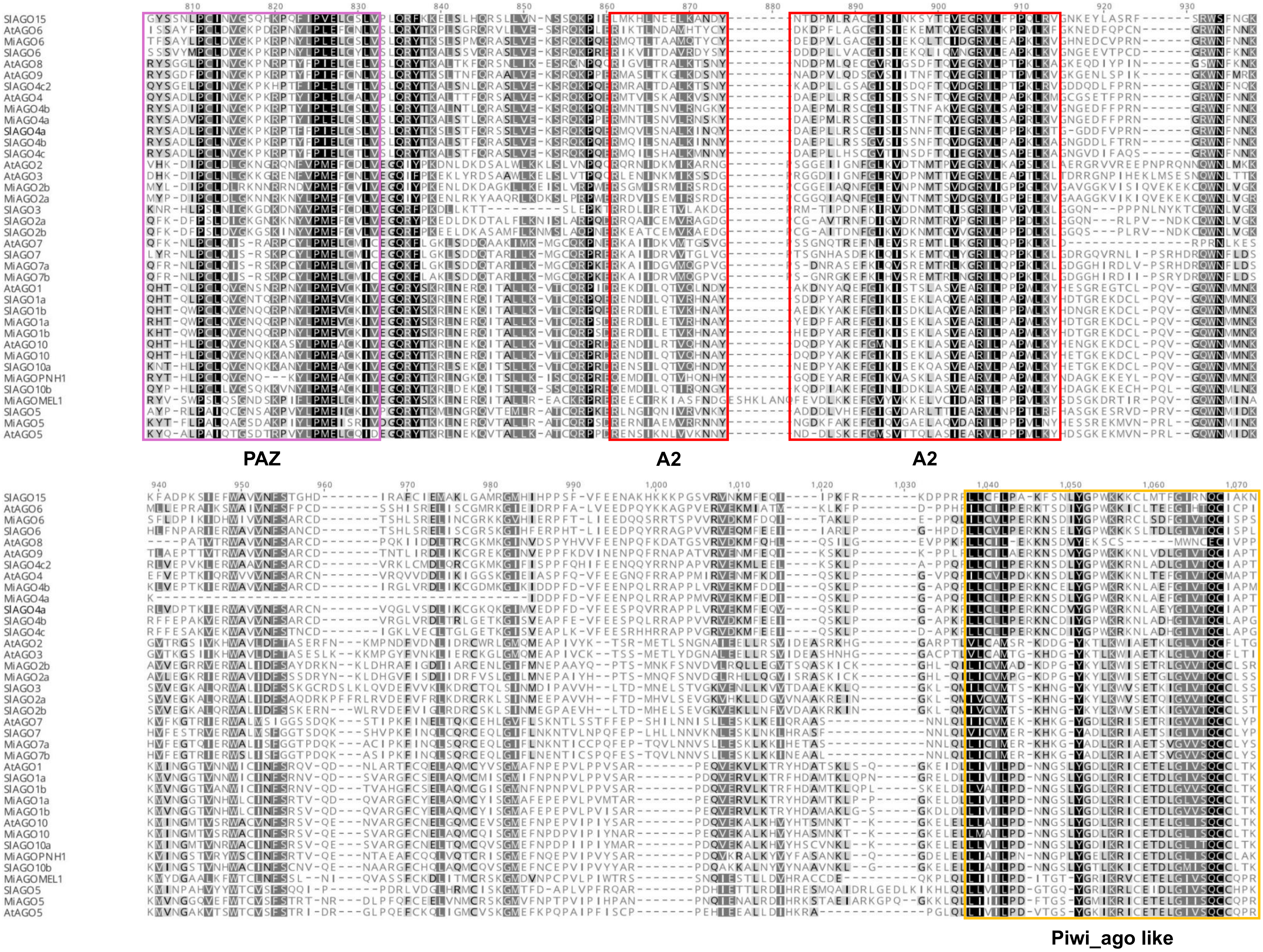
PAZ
A2
A2
Piwi_ago like

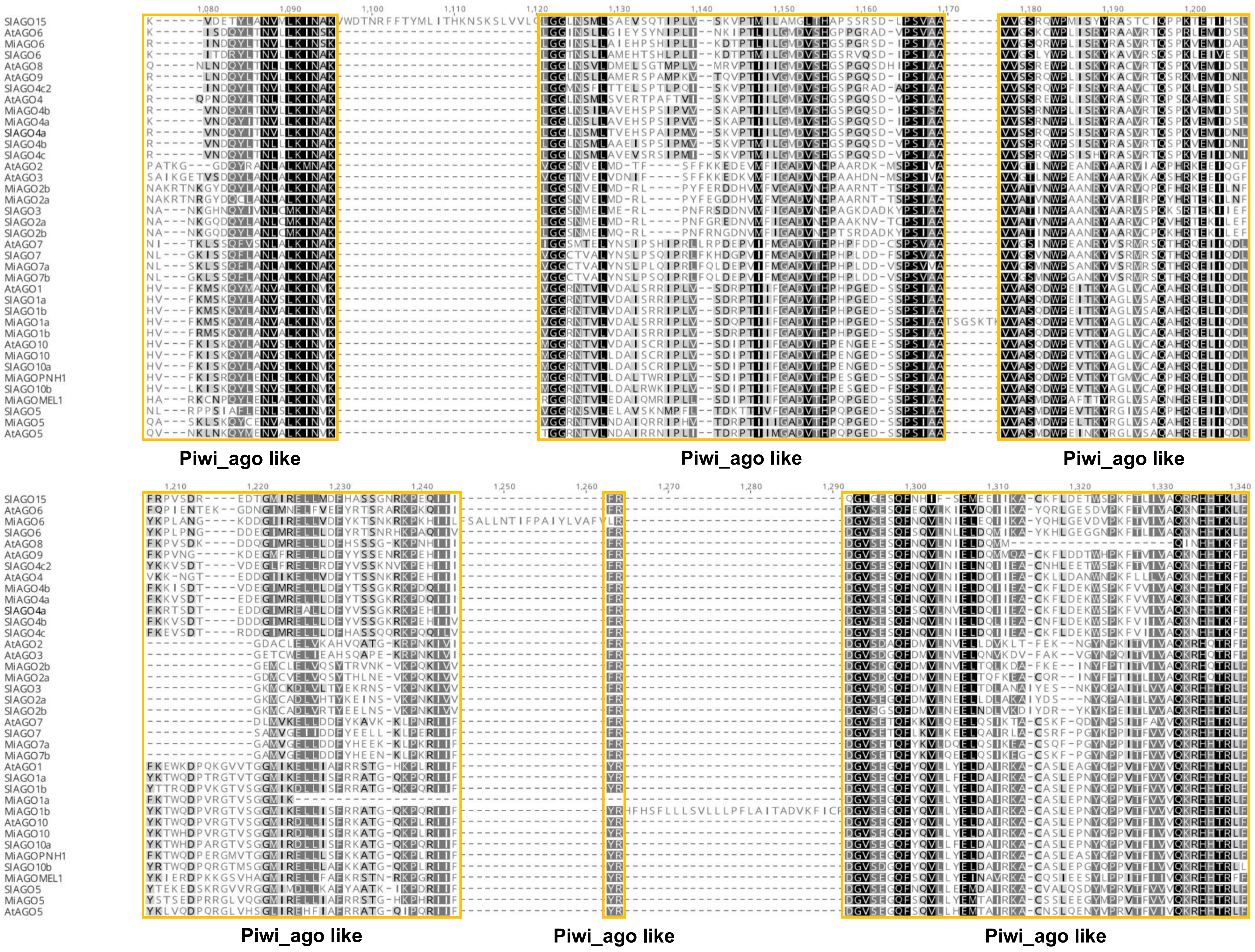
Piwi_ago like
Piwi_ago like
Piwi_ago like
Piwi_ago like
Piwi_ago like
Piwi_ago like

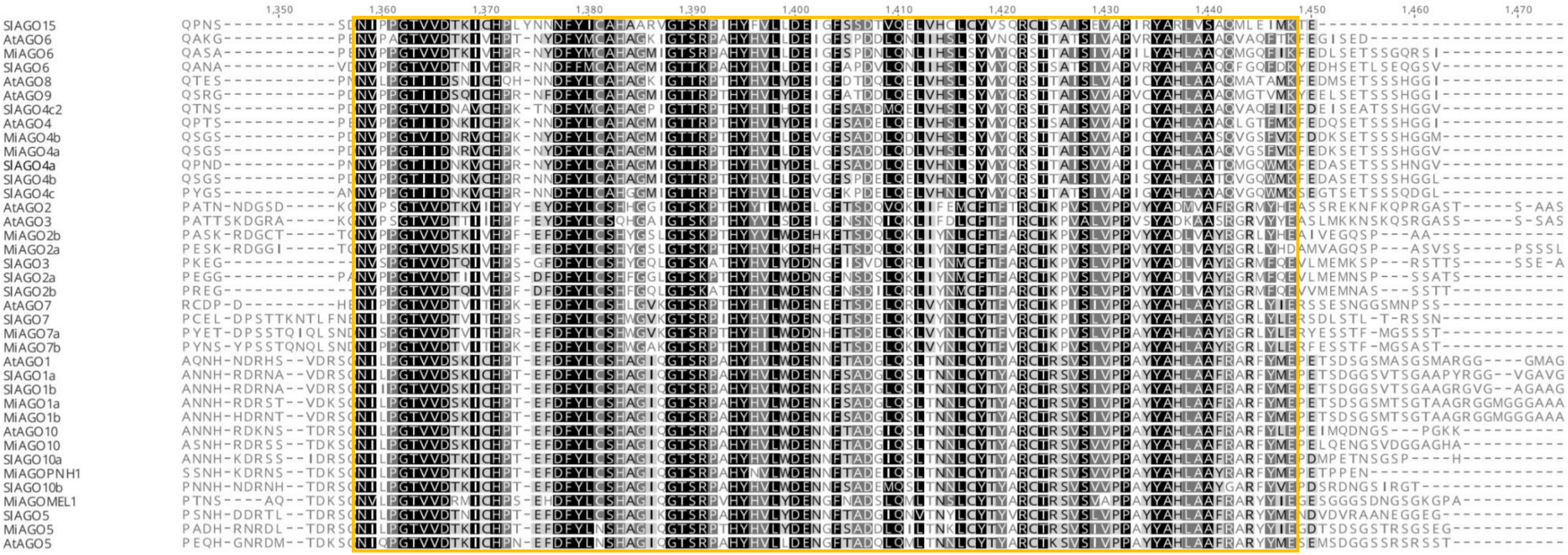
1,350
1,360
1,370
1,380
1,390
1,400
1,410
1,420
1,430
1,440
1,450
1,460
1,470
SlAGO15
AtAGO6
MiAGO6
SlAGO6
AtAGO8
AtAGO9
SlAGO4c2
AtAGO4
MiAGO4b
MiAGO4a
SlAGO4a
SlAGO4b
SlAGO4c
AtAGO2
AtAGO3
MiAGO2b
MiAGO2a
SlAGO3
SlAGO2a
SlAGO2b
AtAGO7
SlAGO7
MiAGO7a
MiAGO7b
AtAGO1
SlAGO1a
SlAGO1b
MiAGO1a
MiAGO1b
AtAGO10
MiAGO10
SlAGO10a
MiAGOPNH1
SlAGO10b
MiAGOMEL1
SlAGO5
MiAGO5
AtAGO5
