# Supplementary material for: Genome-wide identification of gene families related to miRNA biogenesis in Mangifera indica L. and their possible role during heat stress
Source: PeerJ. 2024 Jul 17;12:e17737. doi: 10.7717/peerj.17737 (PMC11260077; doi:10.7717/peerj.17737)
Supplement: Supplemental Information 6 — Colored boxes indicate characteristic domains. [file peerj-12-17737-s006.pdf]

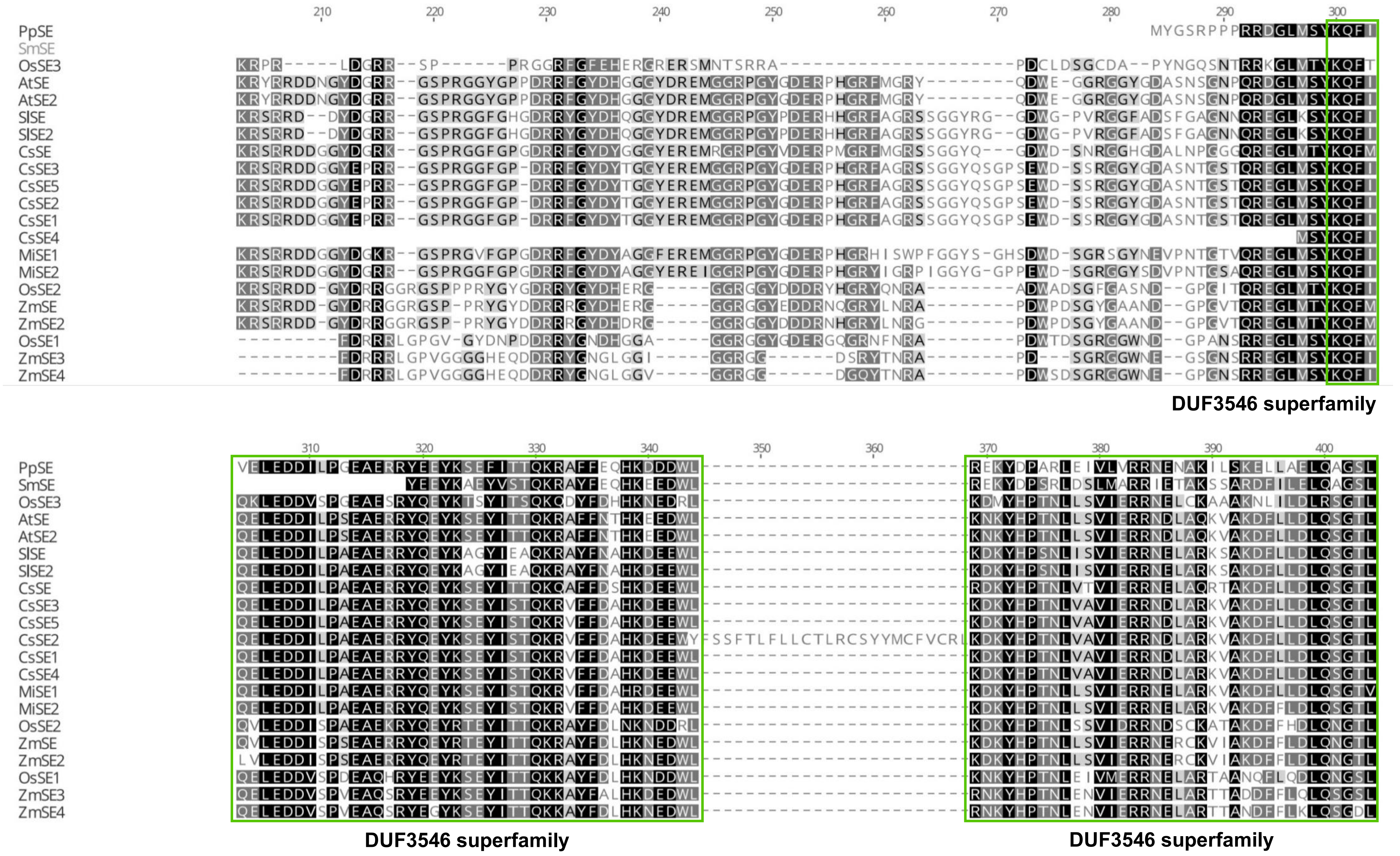
210 220 230 240 250 260 270 280 290 300
PpSE
SmSE
OsSE3
AtSE
AtSE2
SlSE
SlSE2
CsSE
CsSE3
CsSE5
CsSE2
CsSE1
CsSE4
MiSE1
MiSE2
OsSE2
ZmSE
ZmSE2
OsSE1
ZmSE3
ZmSE4
DUF3546 superfamily
310 320 330 340 350 360 370 380 390 400
DUF3546 superfamily
DUF3546 superfamily

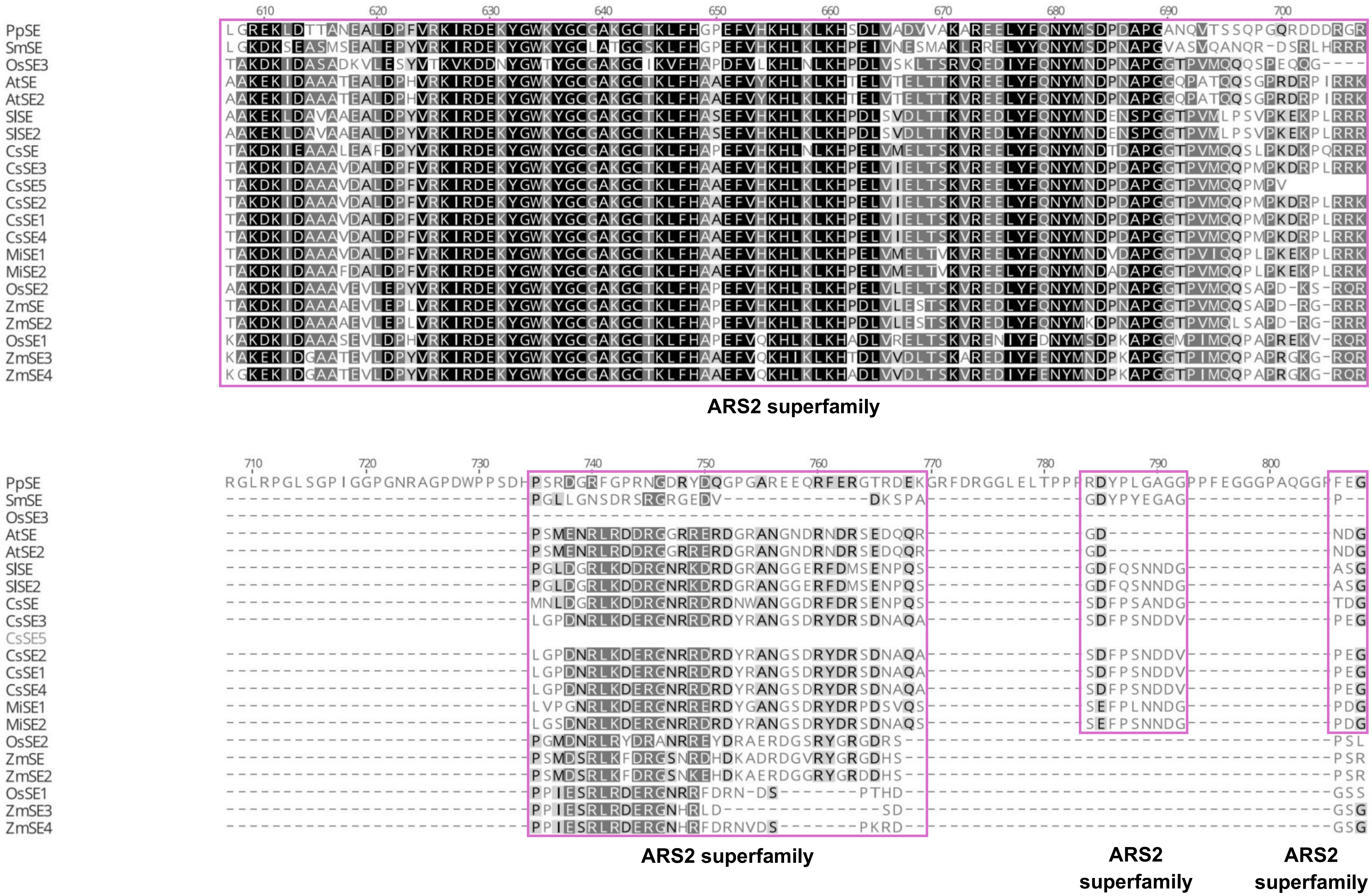
PpSE
SmSE
OsSE3
AtSE
AtSE2
SlSE
SlSE2
CsSE
CsSE3
CsSE5
CsSE2
CsSE1
CsSE4
MiSE1
MiSE2
OsSE2
ZmSE
ZmSE2
OsSE1
ZmSE3
ZmSE4
ARS2 superfamily
ARS2 superfamily
ARS2 superfamily
ARS2 superfamily

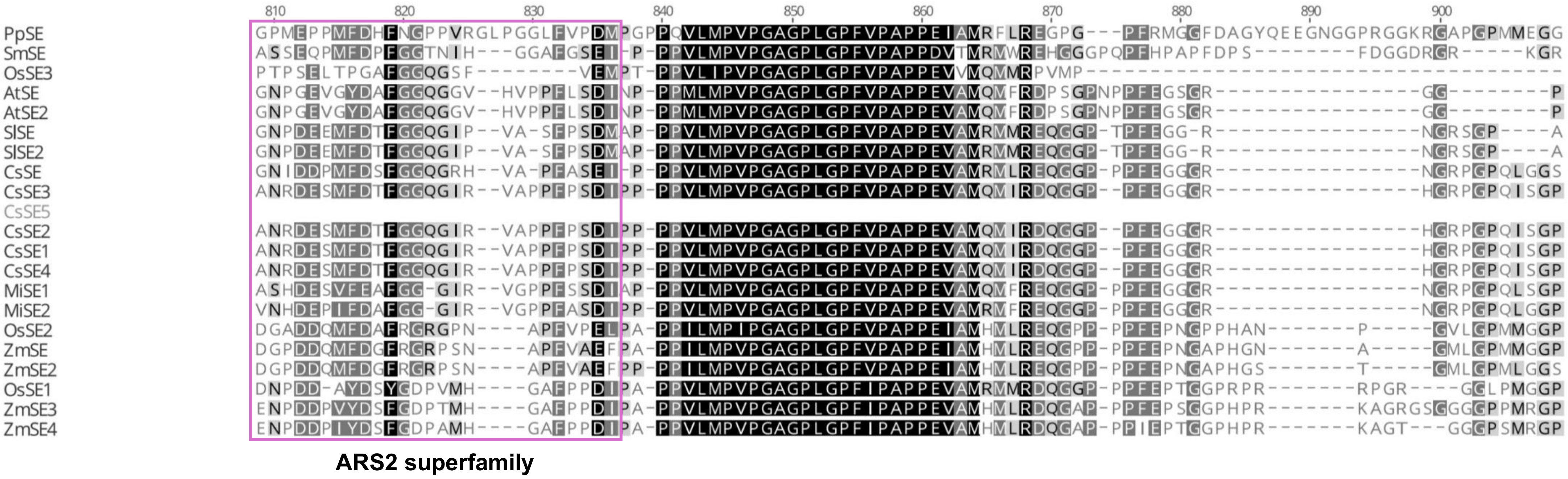
810
820
830
840
850
860
870
880
890
900
PpSE
SmSE
OsSE3
AtSE
AtSE2
SlSE
SlSE2
CsSE
CsSE3
CsSE5
CsSE2
CsSE1
CsSE4
MiSE1
MiSE2
OsSE2
ZmSE
ZmSE2
OsSE1
ZmSE3
ZmSE4
ARS2 superfamily
