# Supplementary material for: Genome-wide identification of gene families related to miRNA biogenesis in Mangifera indica L. and their possible role during heat stress
Source: PeerJ. 2024 Jul 17;12:e17737. doi: 10.7717/peerj.17737 (PMC11260077; doi:10.7717/peerj.17737)
Supplement: Supplemental Information 7 — Colored boxes indicate characteristic domains. [file peerj-12-17737-s007.pdf]

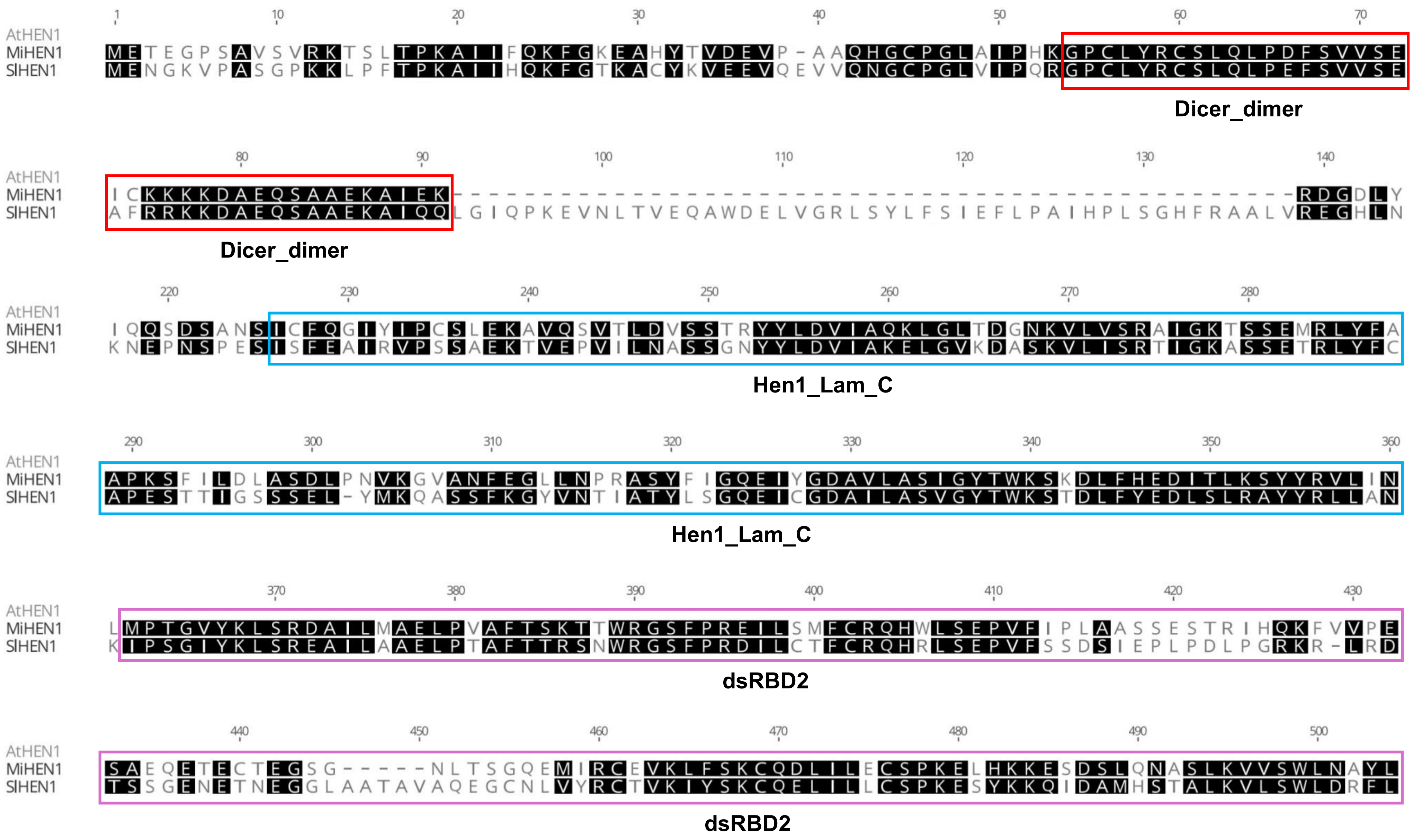
AtHEN1
MiHEN1
SlHEN1
Dicer_dimer
Dicer_dimer
Hen1_Lam_C
Hen1_Lam_C
dsRBD2
dsRBD2

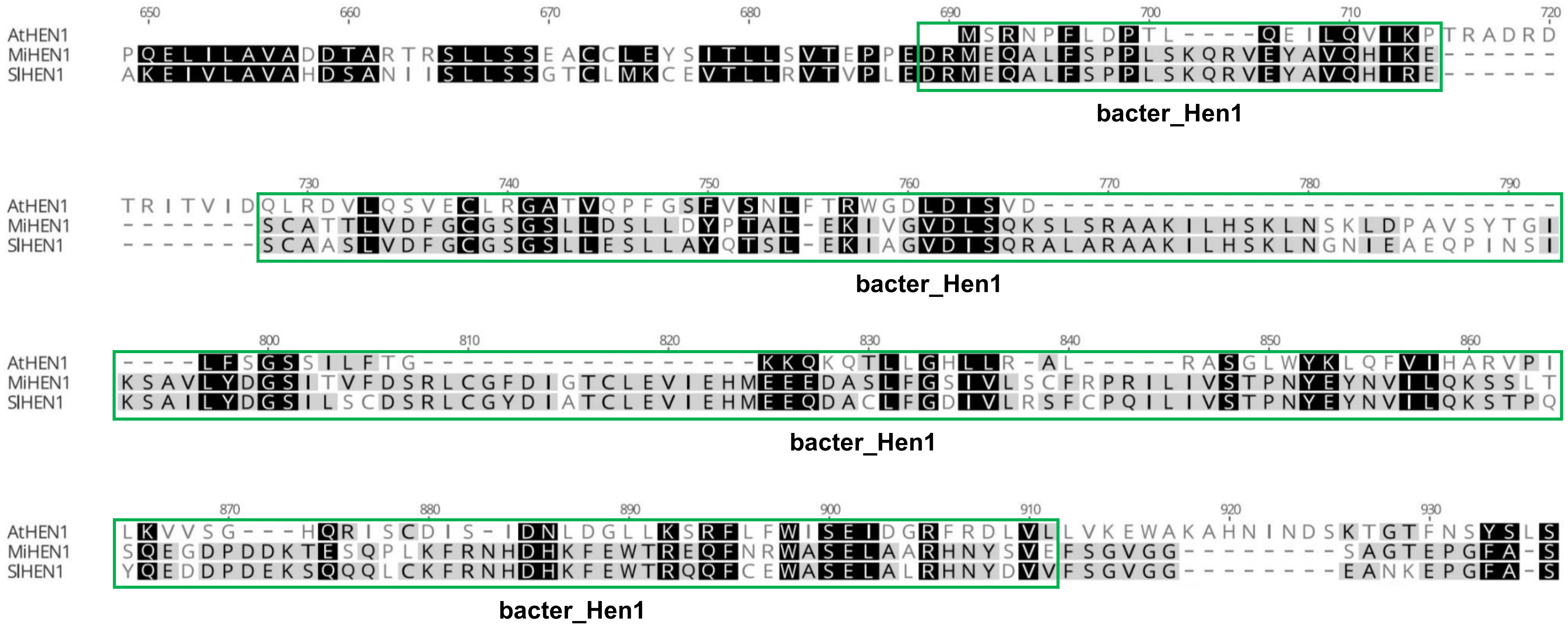
AtHEN1
MiHEN1
SlHEN1
bacter_Hen1
bacter_Hen1
bacter_Hen1
bacter_Hen1
