# Supplementary material for: Genome-wide identification of gene families related to miRNA biogenesis in Mangifera indica L. and their possible role during heat stress
Source: PeerJ. 2024 Jul 17;12:e17737. doi: 10.7717/peerj.17737 (PMC11260077; doi:10.7717/peerj.17737)
Supplement: Supplemental Information 8 — Colored boxes indicate characteristic domains. [file peerj-12-17737-s008.pdf]

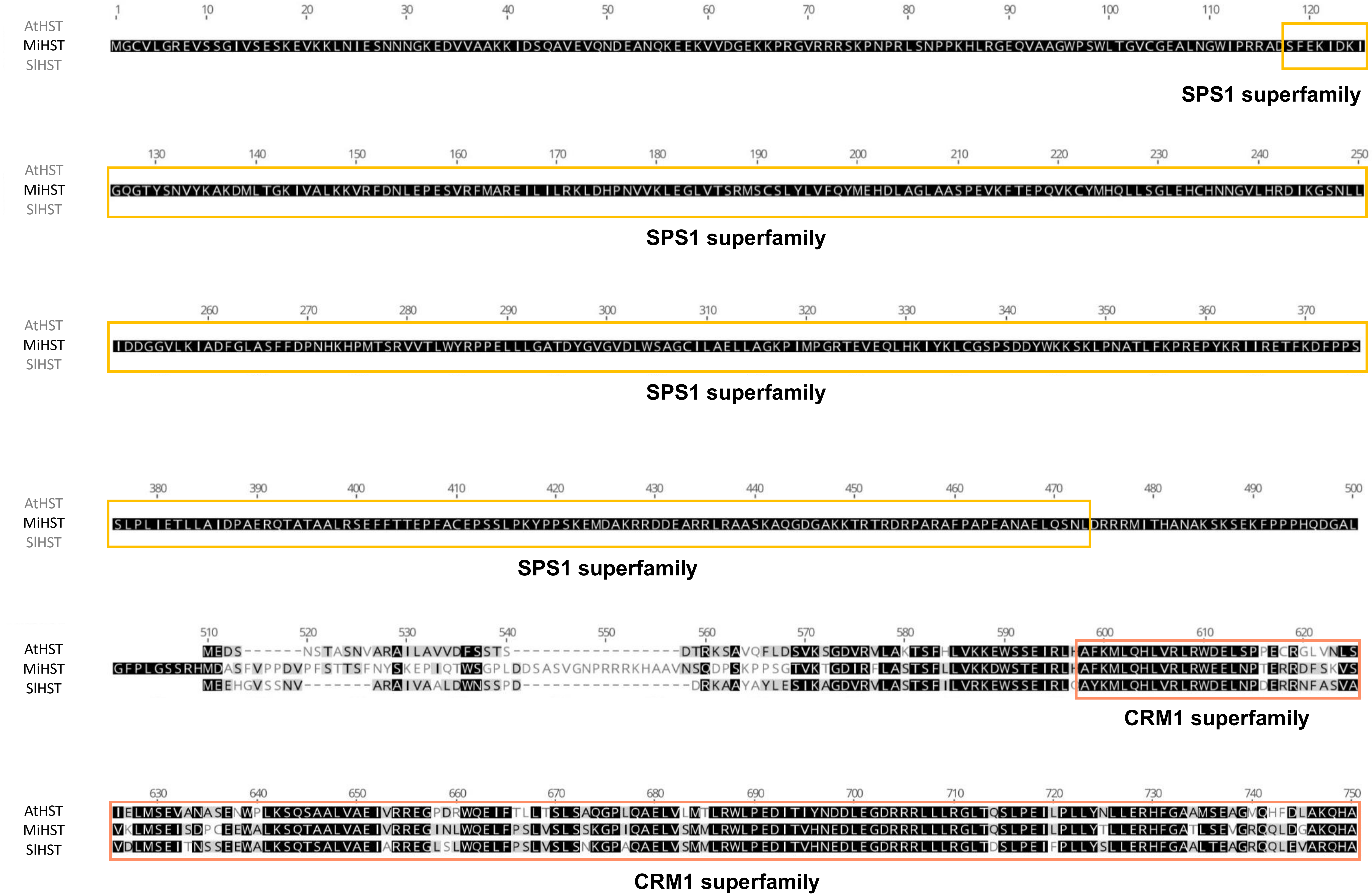

AtHST
MiHST
SlHST
SPS1 superfamily
SPS1 superfamily
SPS1 superfamily
SPS1 superfamily
CRM1 superfamily
CRM1 superfamily

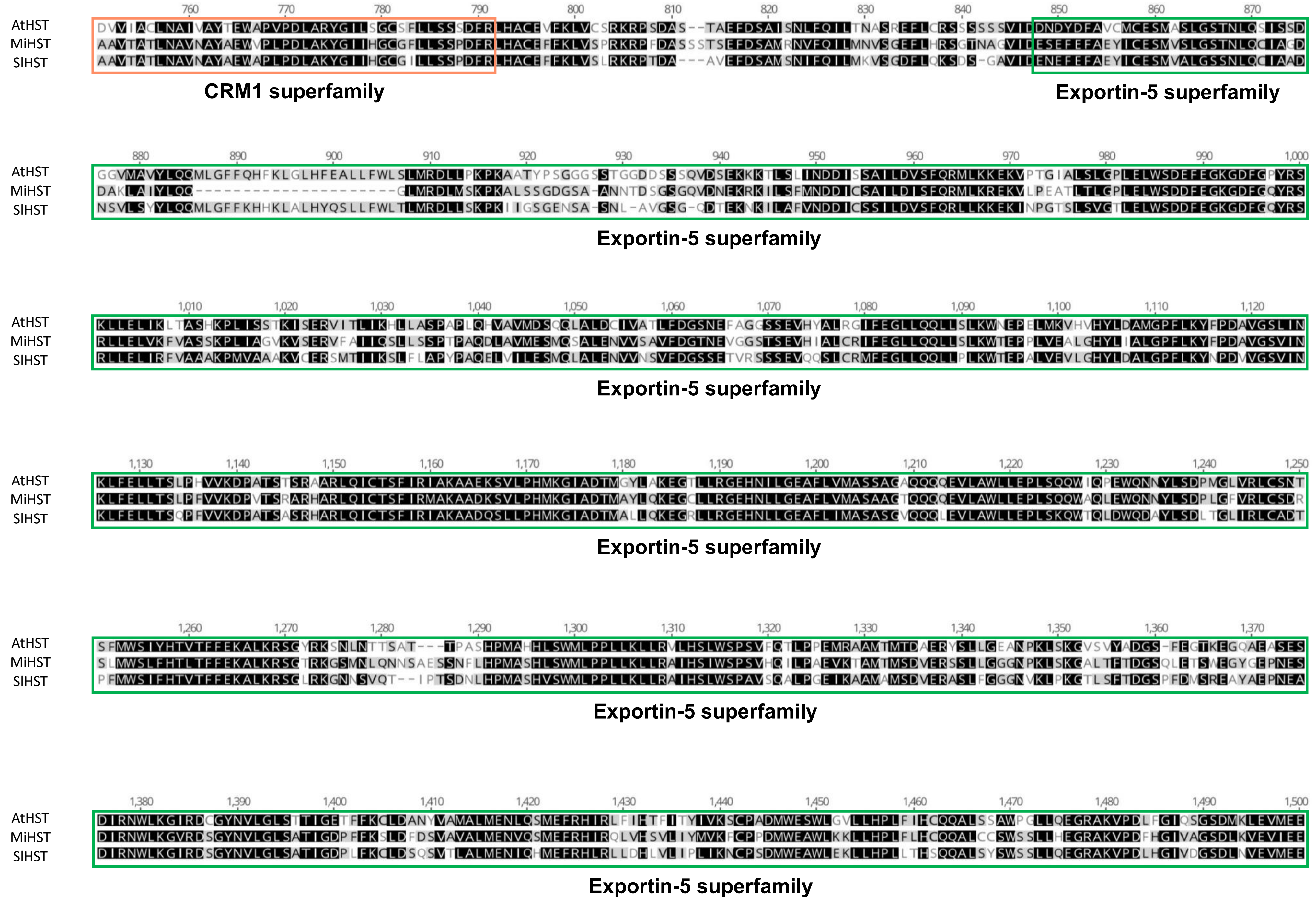
AtHST
MiHST
SlHST
CRM1 superfamily
Exportin-5 superfamily
Exportin-5 superfamily
Exportin-5 superfamily
Exportin-5 superfamily
Exportin-5 superfamily
Exportin-5 superfamily

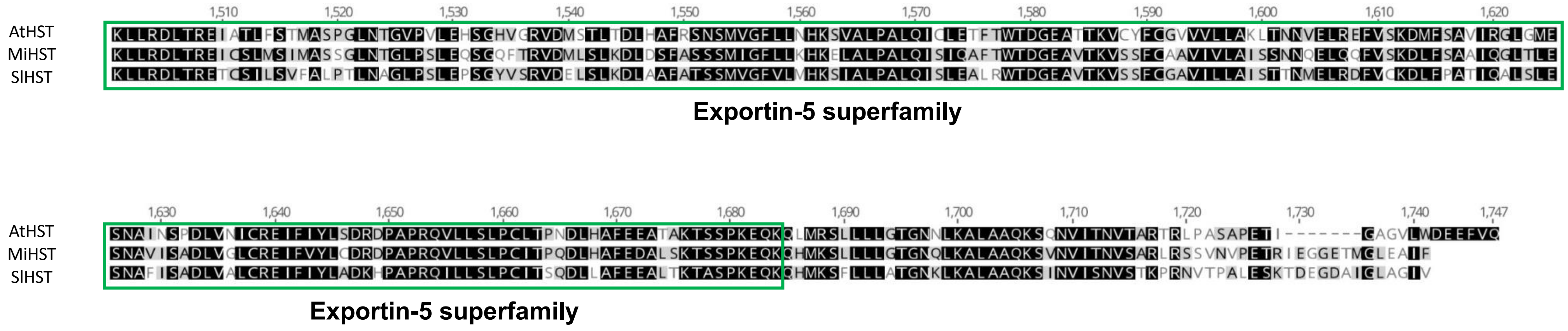
AtHST
MiHST
SlHST
Exportin-5 superfamily
AtHST
MiHST
SlHST
Exportin-5 superfamily
