# Supplementary material for: Genome-wide identification of gene families related to miRNA biogenesis in Mangifera indica L. and their possible role during heat stress
Source: PeerJ. 2024 Jul 17;12:e17737. doi: 10.7717/peerj.17737 (PMC11260077; doi:10.7717/peerj.17737)
Supplement: Supplemental Information 9 — Colored boxes indicate characteristic domains. [file peerj-12-17737-s009.pdf]

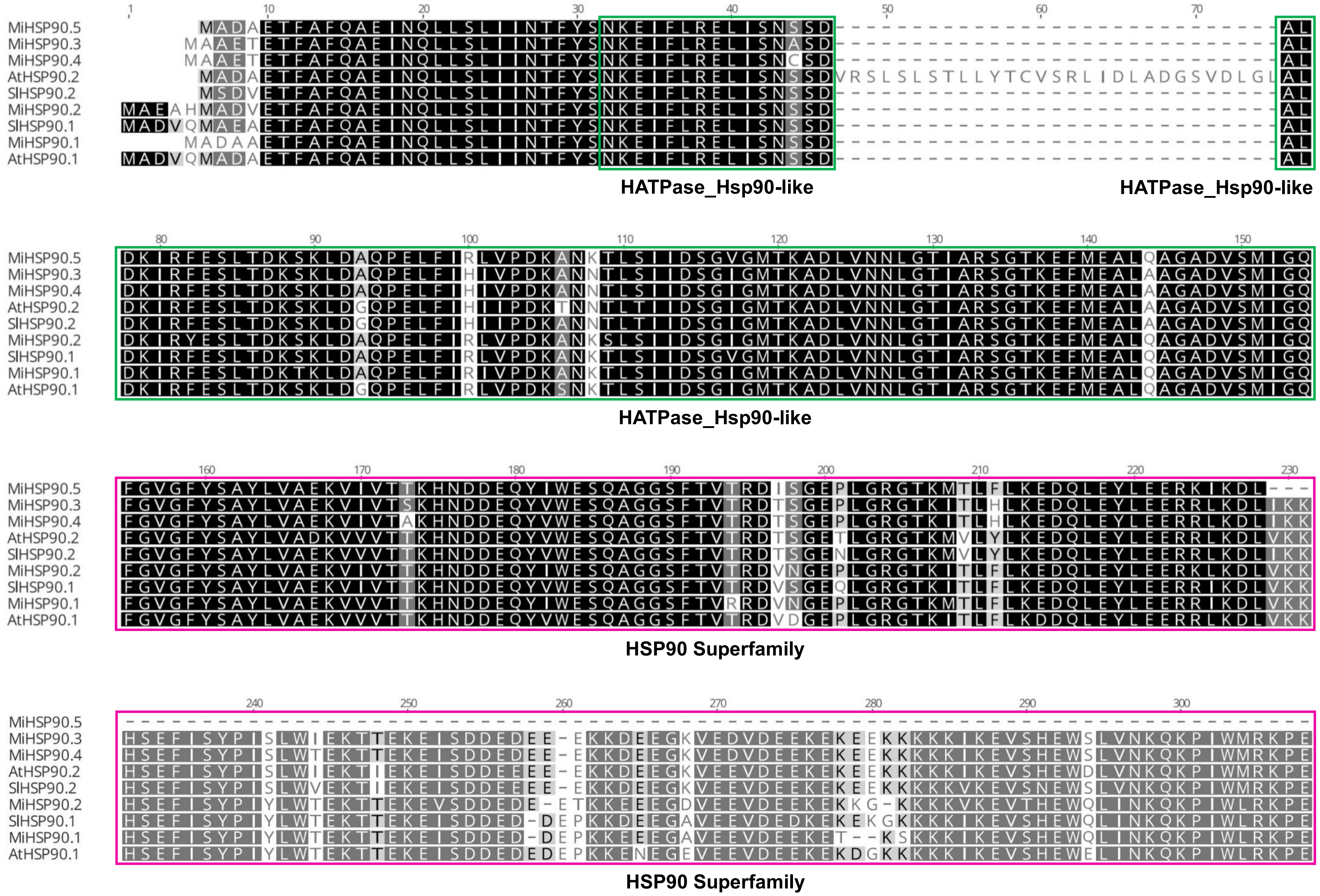
MiHSP90.5
MiHSP90.3
MiHSP90.4
AtHSP90.2
SlHSP90.2
MiHSP90.2
SlHSP90.1
MiHSP90.1
AtHSP90.1
HATPase_Hsp90-like
HATPase_Hsp90-like
HATPase_Hsp90-like
HSP90 Superfamily
HSP90 Superfamily

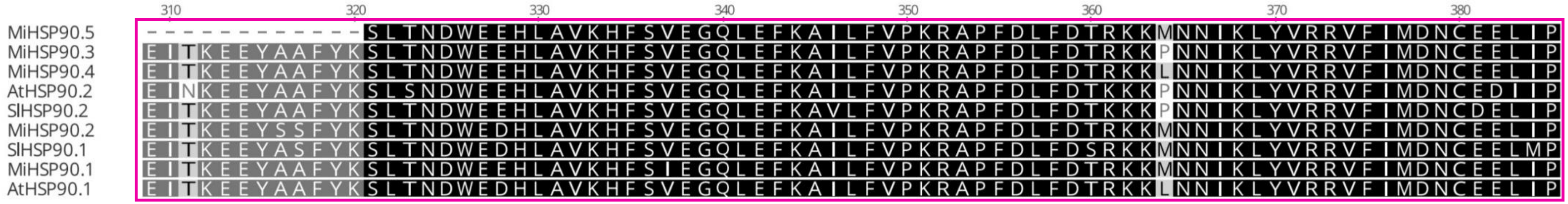


**HSP90 Superfamily**

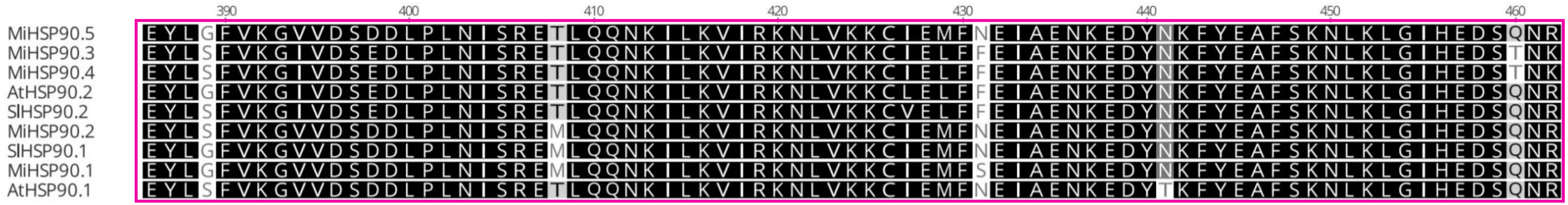


**HSP90 Superfamily**

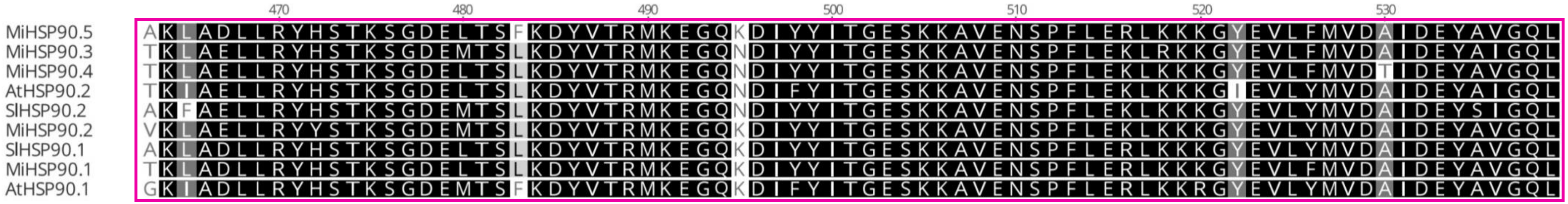


**HSP90 Superfamily**

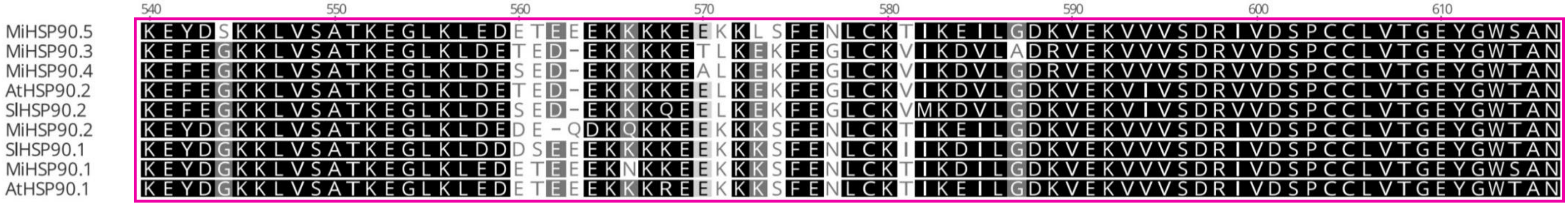


**HSP90 Superfamily**

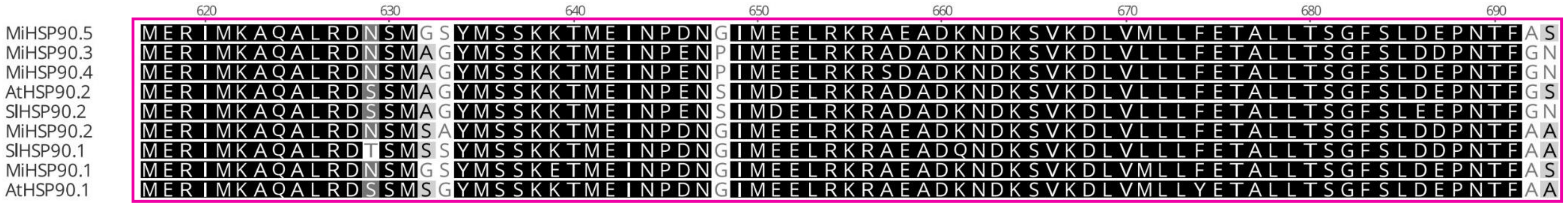


**HSP90 Superfamily**
