# Supplementary material for: Genome-wide identification of gene families related to miRNA biogenesis in Mangifera indica L. and their possible role during heat stress
Source: PeerJ. 2024 Jul 17;12:e17737. doi: 10.7717/peerj.17737 (PMC11260077; doi:10.7717/peerj.17737)
Supplement: Supplemental Information 10 [file peerj-12-17737-s010.docx]

**Table S1. Proteins used for the Phylogenetic analysis and their accession numbers**

| **Specie** | **Protein** | **GenBank Accession** |
| --- | --- | --- |
|  | **DICER-LIKE** |  |
| *Citrus sinensis* | CsDCL1 | KAH9787193.1 |
|  | CsDCL2 | XP_006480489.1 |
|  | CsDCL3 | XP_024953169.1 |
|  | CsDCL4 | XP_006473957.1 |
| *Cucumis sativus* | CsDCL | XP_011650612.1 |
|  | CsDCL2 | XP_004139098.1 |
| *Solanum lycopersicum* | SlDCL1 | AGN12837.1 |
|  | SlDCL2a | AMS34005.1 |
|  | SlDCl2b | AMS34006.1 |
|  | SlDCl2c | AMS34007.1 |
|  | SlDCl2d | AMS34008.1 |
|  | SlDCl3 | AHV82113.1 |
| *Arabidopsis thaliana* | AtDCL1 | Q9SP32.2 |
|  | AtDCL2 | Q3EBC8.2 |
|  | AtDCL3 | Q9LXW7.2 |
|  | AtDCL4 | P84634.2 |
| *Oryza sativa* | OsDCL1 | Q8LMR2.1 |
|  | OsDCL2A | NP_001389112.1 |
|  | OsDCL2B | NP_001409657.1 |
|  | OsDCL3A | Q5N870.1 |
|  | OsDCL3B | Q7XD96.2 |
|  | OsDCL4 | NP_001389326.1 |
| *Physcomitrium patens* | PpDCL1a | XP_024368603.1 |
|  | PpDCl1b | XP_024363919.1 |
|  | PpDCL1c | XP_024363918.1 |
|  | PpDCL3A | XP_024361919.1 |
|  | PpDCL4 | XP_024366627.1 |
| *Selaginella moellendorffii* | SmDCL1 | XP_024538863.1 |
|  | SmDCL2 | XP_002982062.2 |
|  | SmDCL3A | XP_024530206.1 |
|  | SmDCL4 | XP_024527017.1 |
|  | **ARGONAUTE** |  |
| *Citrus sinensis* | CsAGO1 | XP_006478119.1 |
|  | CsAGO2 | XP_006468440.1 |
|  | CsAGO4 | XP_006470434.1 |
|  | CsAGO5 | XP_006484939.1 |
|  | CsAGO7 | XP_006483096.1 |
|  | CsAGO10 | XP_006489474.1 |
| **Specie** | **Protein** | **GenBank Accession** |
|  | CsAGO16 | XP_006469598.1 |
|  | CsAGOMEL1 | XP_006485141.1 |
| *Cucumis sativus* | CsAGOPNH1 | XP_004146290.1 |
|  | CsAGO4 | XP_011653531.1 |
|  | CsAGO5 | XP_031741995.1 |
|  | CsAGO16 | XP_004140126.1 |
|  | CsAGO7 | XP_004135160.1 |
|  | CsAGO10 | XP_004134114.1 |
| *Solanum lycopersicum* | SlAGO1A | NP_001266057.1 |
|  | SlAGO3 | NP_001274720.1 |
|  | SlAGO4A | NP_001266156.1 |
|  | SlAGO4 | XP_019069922.1 |
|  | SlAGO5 | NP_001265878.1 |
|  | SlAGO6 | NP_001266273.1 |
|  | SlAGO7 | NP_001266209.1 |
|  | SlAGO10 | NP_001266268.1 |
|  | SlAGO18 | XP_010326816.1 |
| *Arabidopsis thaliana* | AtAGO1 | NP_175274.1 |
|  | AtAGO2 | NP_174413.2 |
|  | AtAGO3 | Q9SHF2.1 |
|  | AtAGO4 | Q9ZVD5.2 |
|  | AtAGO5 | Q9SJK3.2 |
|  | AtAGO6 | O48771.2 |
|  | AtAGO7 | Q9C793.1 |
|  | AtAGO8 | Q3E984.1 |
|  | AtAGO9 | Q84VQ0.2 |
|  | AtAGO10 | Q9XGW1.1 |
| *Oryza sativa* | OsAGO1A | NP_001403746.1 |
|  | OsAGO2 | NP_001406794.1 |
|  | OsAGO3 | NP_001406795.1 |
|  | OsAGO4A | NP_001415038.1 |
|  | OsAGO7 | NP_001405268.1 |
|  | OsAGO11 | Q10F39.2 |
|  | OsAGO18 | NP_001409080.1 |
|  | OsAGOPNH1 | NP_001411224.1 |
| *Physcomitrium patens* | PpAGO1 | XP_024401595.1 |
|  | PpAGO4 | XP_024384187.1 |
|  | PpAGO7 | XP_024400268.1 |
|  | PpAGO9 | XP_024373037.1 |
|  | PpAGO10 | XP_024403748.1 |
|  | PpAGO16 | XP_024356712.1 |
| **Specie** | **Protein** | **GenBank Accession** |
| *Selaginella moellendorffii* | SmAGO1 | XP_024539711.1 |
|  | SmAGO10 | EFJ24991.1 |
|  | SmAGOPNH1 | XP_024534515.1 |
|  | **HUA ENHANCER 1** |  |
| *Citrus sinensis* | CsHEN1 | KDO75225.1 |
| *Cucumis sativus* | CsHEN1 | XP_004135729.1 |
| *Solanum lycopersicum* | SlHEN1 | XP_004233884.1 |
| *Arabidopsis thaliana* | AtHEN1 | NP_001318386.1 |
| *Oryza sativa* | OsHEN1 | KAB8104419.1 |
| *Physcomitrium patens* | PpHEN1 | PNR61580.1 |
| *Selaginella moellendorffii* | SmHEN1 | XP_024539336.1 |
|  | **HASTY** |  |
| *Citrus sinensis* | CsHST1 | KAH9754448.1 |
| *Cucumis sativus* | CsHST1 | XP_031737031.1 |
| Solanum lycopersicum | SlHST1 | XP_004230145.1 |
| *Arabidopsis thaliana* | AtHST1 | NP_187155.2 |
| *Oryza sativa* | OsHST1 | XP_015611561.1 |
| *Physcomitrium patens* | PpHST1 | XP_024381270.1 |
| *Selaginella moellendorffii* | SmHST1 | XP_024534315.1 |
| *Zea mays* | ZmHST1 | AQK91595.1 |
|  | **HYPONASTIC LEAVES 1** |  |
| *Citrus sinensis* | CsHYL1 | KAH9756189.1 |
|  | CsHYL1 | KDO81203.1 |
| *Cucumis sativus* | CsHYL1 | XP_011649689.1 |
| *Solanum lycopersicum* | SlHYL1 | XP_004238005.1 |
| *Arabidopsis thaliana* | AtHYL1 | NP_563850.1 |
| *Oryza sativa* | OsHYL1 | NP_001415072.1 |
| *Physcomitrium patens* | PpHYL1 | PNR35725.1 |
| *Selaginella moellendorffii* | SmHYL1 | EFJ16766.1 |
| *Zea mays* | ZmHYL1 | ONM61314.1 |
|  | **SERRATE** |  |
| *Citrus sinensis* | CsSE | KAH9790382.1 |
|  | CsSE | KDO66401.1 |
|  | CsSE | KDO66403.1 |
|  | CsSE | KDO66405.1 |
|  | CsSE | KDO66404.1 |
| *Cucumis sativus* | CsSE | XP_004144078.1 |
| *Solanum lycopersicum* | SlSE | XP_004228474.1 |
| *Arabidopsis thaliana* | AtSE | NP_565635.1 |
| *Oryza sativa* | OsSE | XP_015649800.1 |
|  | OsSE | XP_025882281.1 |
| **Specie** | **Protein** | **GenBank Accession** |
| *Oryza sativa* | OsSE | XP_015623289.1 |
| *Physcomitrium patens* | PpSE | XP_024398982.1 |
| *Selaginella moellendorffii* | SmSE | EFJ30030.1 |
| *Zea mays* | ZmSE | NP_001145860.1 |
|  | ZmSE | AQK81170.1 |
|  | ZmSE | NP_001292800.1 |
|  | ZmSE | ONM03103.1 |
|  | **Heat Shock Protein 90** |  |
| *Citrus sinensis* | CsHSP80 | XP_006452664.1 |
| *Cucumis sativus* | CsHSP90 | XP_004152644.1 |
| *Solanum lycopersicum* | SlHSP90 | NP_001308492.1 |
| *Arabidopsis thaliana* | AtHSP90.1 | AM91104.1 |
|  | AtHSP90.2 | NP_200414.1 |
|  | AtHSP90.3 | NP_200412.1 |
|  | AtHSP90.4 | NP_200411.1 |
|  | AtHSP90.5 | NP_194150.1 |
|  | AtHSP90.6 | NP_178487.1 |
|  | AtHSP90.7 | AAF13098.1 |
| *Oryza sativa* | AtHSP90 | BAA90487.1 |
| *Physcomitrium patens* | AtHSP90.2 | XP_024396882.1 |
| *Selaginella moellendorffii* | AtHSP80.1 | XP_002976693.1 |
| *Zea mays* | AtHSP81.1 | NP_001170475.1 |
