# Supplementary material for: Genome-wide identification of gene families related to miRNA biogenesis in Mangifera indica L. and their possible role during heat stress
Source: PeerJ. 2024 Jul 17;12:e17737. doi: 10.7717/peerj.17737 (PMC11260077; doi:10.7717/peerj.17737)
Supplement: Supplemental Information 11 [file peerj-12-17737-s011.docx]

| Primer | Sequence (5’-3’) |
| --- | --- |
| AGO6_Fw | CGGCCCTGTGATTGATTT |
| AGO6_Rv | GCGGTGCCTTGTCTTAAT |
| AGO1b_Fw | GGGCCACTTCCATTTATCTC |
| AGO1b_Rv | CACAACTCTGAATTCCCTCTC |
| AGO4b_Fw | CAGGGATCCGTATGCAATAG |
| AGO4b_Rv | CAGGGTTTCTCACTCAATCC |
| DCL1_Fw | AGTAGAAGGGCTCGAGTTAG |
| DCL1_Rv | ACGTGGCCTCTTCCTATTA |
| DCL3_Fw | TGGACTGCTGGGAGAAA |
| DCL3_Rv | CCATCTCTAGCCTCAGGAA |
| HEN1_Fw | TGGTAAGGAGGCACACTAT |
| HEN1_Rv | GCAAACTGCAGCGATAAAG |
| HST_Fw | GTGAAGTTGGTAGGCAACA |
| HST_Rv | AGCAAGATCAGGCAAAGG |
| GAPDH_Fw | GTGGCTGTTAACGATCCCTT |
| GAPDH_Rv | GTGACTGGCTTCTCATCGAA |
| HSP90_Fw | AAGCTTGTCTCTGCAACC |
| HSP90_Rv | CACAGACCCTCAAACTTCTC |

**Table S2. List of primers used for qPCR reactions**
