# Supplementary material for: Genome-wide identification of gene families related to miRNA biogenesis in Mangifera indica L. and their possible role during heat stress
Source: PeerJ. 2024 Jul 17;12:e17737. doi: 10.7717/peerj.17737 (PMC11260077; doi:10.7717/peerj.17737)
Supplement: Supplemental Information 5 — Colored boxes indicate characteristic domains. [file peerj-12-17737-s005.pdf]

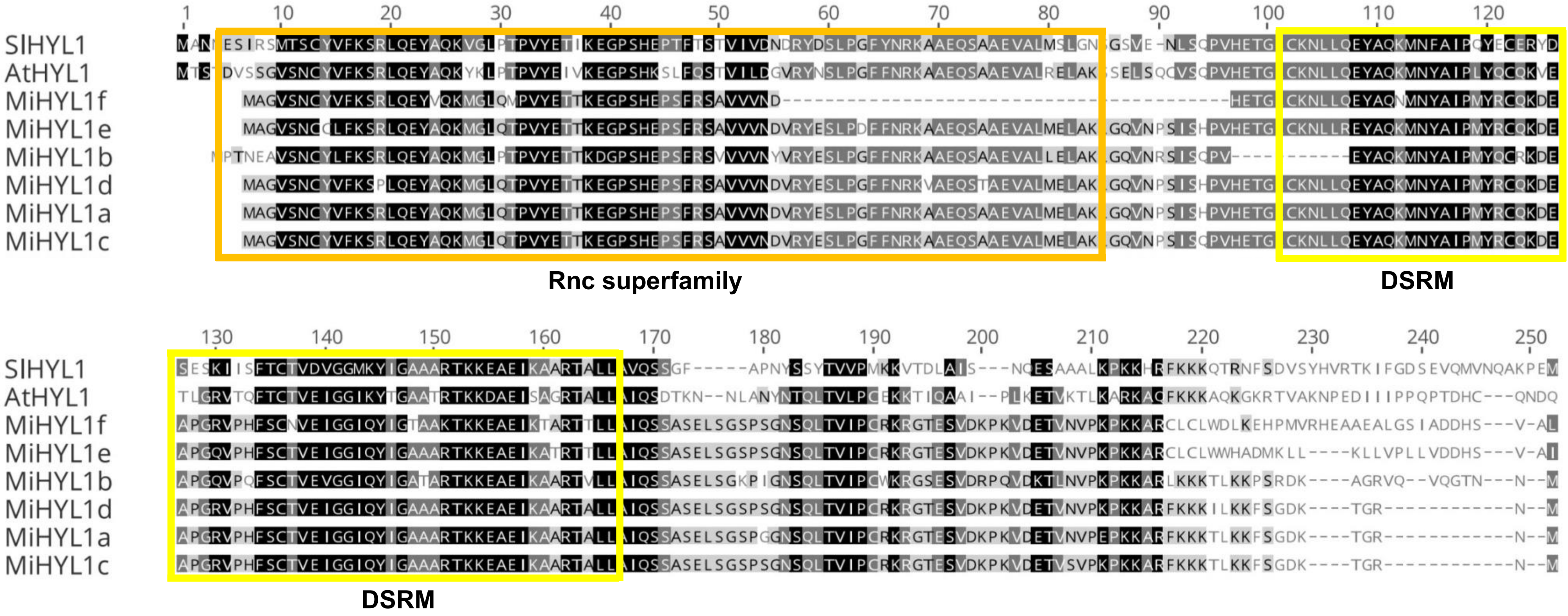
SlHYL1
AtHYL1
MiHYL1f
MiHYL1e
MiHYL1b
MiHYL1d
MiHYL1a
MiHYL1c
Rnc superfamily
DSRM
DSRM
